# How HIV exploits T cells in the endometrium

**DOI:** 10.7554/eLife.58169

**Published:** 2020-05-26

**Authors:** Marta Rodriguez-Garcia

**Affiliations:** Department of Immunology, Tufts University School of MedicineBostonUnited States

**Keywords:** HIV in women, HIV, female reproductive tract, viruses, T cells, mucosa, Human, Virus

## Abstract

Immune cells in the endometrium are targeted by HIV and re-programmed to allow them to survive and spread the virus throughout the body.

**Related research article** Ma T, Luo X, George AF, Mukherjee G, Sen N, Spitzer TL, Giudice LC, Greene WC, Roan NR. 2020. HIV efficiently infects T cells from the endometrium and remodels them to promote systemic viral spread. *eLife* **9**:e55487. doi: 10.7554/eLife.55487

HIV was discovered in the 1980s, but all attempts to develop a vaccine since then have failed. Antiretroviral drugs have been used to help prevent HIV infection, but with limited efficacy in women (Marrazzo, 2018). This problem is particularly important in endemic areas: in sub-Saharan Africa, for example, around 80% of new HIV infections in people between the age of 15 and 19 happen in women, which means that a young woman gets infected every three minutes (UNAIDS, 2018). Sexual transmission is the main mechanism for HIV acquisition: the virus enters the body through the genital tract, establishes genital infection, and then spreads to other parts of the body. Prevention therefore requires measures that stop the virus from infecting immune cells in the genital tract. Learning more about the cells that get infected, and about how the virus spreads from the genital tract to the rest of the body, are areas of intense investigation.

The female genital tract is divided into different anatomical compartments (Figure 1), each with different functions, and the immune cells in these compartments have to balance their role in reproduction with their role in protecting the body against infection. The human endometrium is particularly unique as the type of immune cells present and their functions are strongly regulated every month by sex hormones that optimize conditions for pregnancy (Wira et al., 2015). When talking about sexual transmission of HIV, most people think that infection happens in the vagina as early studies in non-human primates identified groups of HIV-infected cells in the lower tract (Zhang et al., 1999). Most subsequent studies in humans also focused on the lower tract, due in part to easier access to human genital samples, and for a long time the role of the upper tract was overlooked. However, more recent studies in non-human primates demonstrated that infection can occur anywhere in the genital tract (Stieh et al., 2014). Now, in eLife, Nadia Roan (the Gladstone Institute and UC San Francisco) and colleagues – including Tongcui Ma as first author – report the results of in vitro experiments on immune cells called T cells which show that the endometrium in the upper tract is also a potential portal of entry for HIV (Ma et al., 2020).

**Figure 1.**
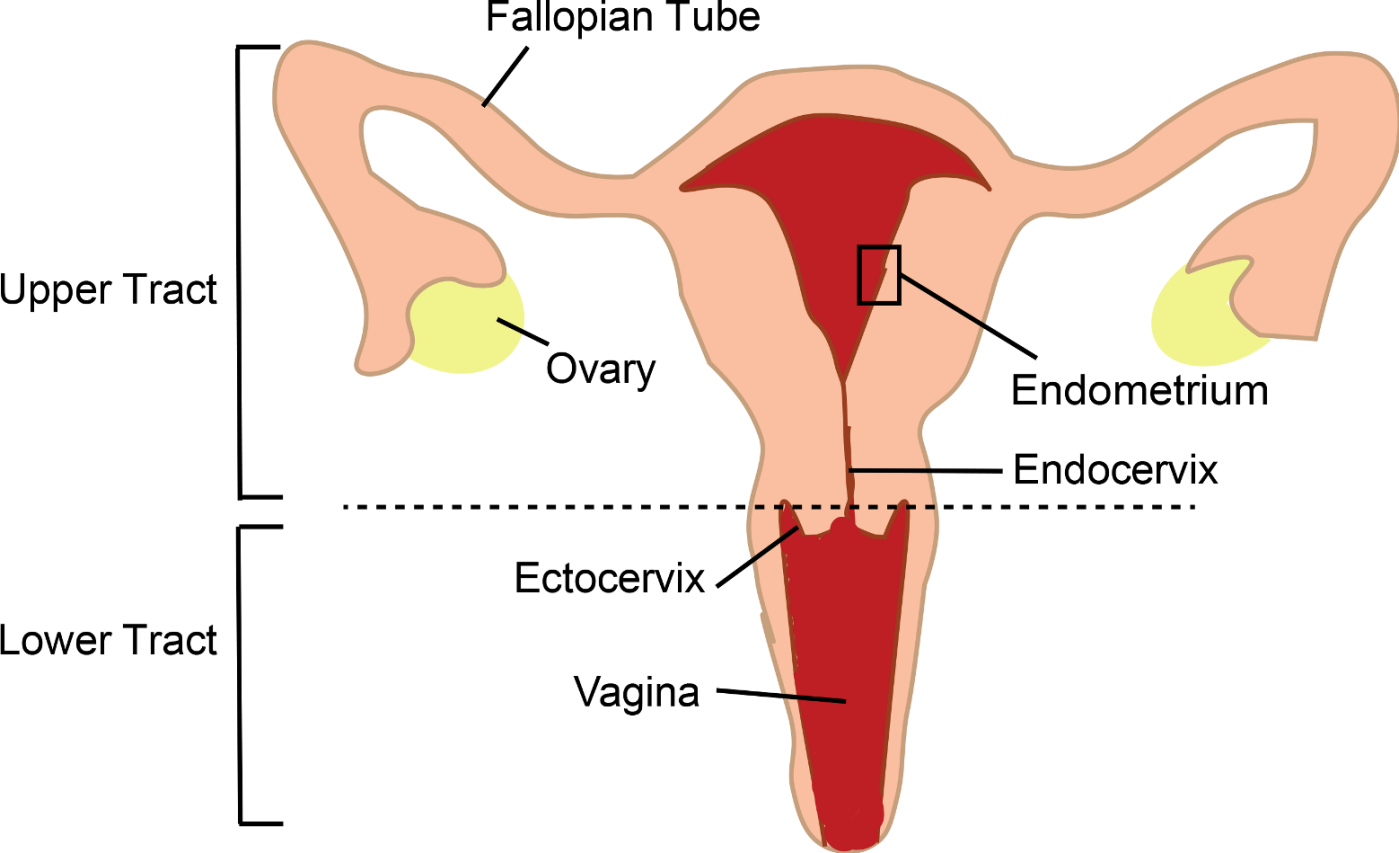
The female genital tract and HIV infection. The lower tract includes the vagina and ectocervix; the upper tract includes the endocervix, endometrium, Fallopian tubes and ovaries. Semen enters thought the lower tract and travels to the Fallopian tubes where egg fertilization occurs; the fertilized egg then travels to the endometrium, where implantation and pregnancy take place. The immune cells of the genital tract that coordinate pregnancy and protect against infections are themselves the initial targets for infection by HIV in women. Previously research in this area has mostly focused on immune cells in the transitional zone between the ectocervix and endocervix. The work of Ma et al. highlights the importance of immune cells in the endometrium.

The latest work differs from previous studies in a number of ways. First, it focuses on the endometrium while, as mentioned above and with just a few exceptions, previous research has focused on the lower tract. Second, the methodology is optimized to closely model heterosexual transmission. In vivo, HIV is transported into the genital tract by semen and it is known that only some viruses (called transmitted founder viruses) have the ability to initiate infection (Keele et al., 2008). To mimic these aspects in vitro, Ma et al. performed infections in the presence of a selected component of semen and using a transmitted founder virus. Methodological details are extremely important when comparing studies of HIV infection in vitro, as seminal components and viral strains influence infectivity and cell selection. Finally, the researchers used extensive cell phenotyping, combined with bioinformatics tools, to provide the most comprehensive characterization of HIV-infected T cells in the genital tract to date.

Ma et al. obtained endometrial biopsies and blood from the same women, performed in vitro HIV infection, and compared T cells side-by-side. In the absence of in vitro pre-activation (a common method used to increase infection levels), they found that T cells from the endometrium were more efficiently infected by HIV than blood T cells, whether the blood T cells were activated or not. Phenotypical characterization confirmed that compared to blood T cells, the endometrial T cells were predominantly a type of T cell called an effector memory T cell, and that they expressed higher levels of CCR5, the co-receptor used by HIV to enter genital cells (Saba et al., 2010). Next, using bioinformatics tools, they showed that just one subset of blood T cells were preferentially infected, whereas multiple subsets of T cells from the endometrium were infected.

The subsets preferentially infected included Th1, Th2 and Tfh. Previous studies of the lower tract in women and non-human primates had found that a different subset, Th17, was a preferential target for HIV (Rodriguez-Garcia et al., 2014; Joag et al., 2016; Stieh et al., 2016). Th17 cells are abundant in tissues colonized by microbiota, where they mediate immune protection and help maintain the integrity of epithelial barriers, but they are only found in small numbers in the human endometrium, as reported by the present author and colleagues in 2014 (Rodriguez-Garcia et al., 2014), and confirmed by Ma et al. The level of Th17 cells in the endometrium may be low because these cells have been implicated in recurrent pregnancy loss (Lee et al., 2012). The unique conditions in the human endometrium influence susceptibility to infection and cannot be fully captured by animal models, so studies like that of Ma et al. are necessary to understand HIV infection in women.

Crucially, the study demonstrates that HIV remodels the endometrial T cells that get infected in a way that maintains and spreads infection. The researchers identified cell modifications that would impair antigen presentation, increase cell survival, and facilitate dissemination from the endometrium to draining lymph nodes. These findings provide several potential markers that could be targeted in future strategies to limit the survival and spread of cells infected with HIV. Consistent with previous reports (Joag et al., 2016; Cantero-Pérez et al., 2019; Cavrois et al., 2019), Ma et al. observed that HIV targeted T cells expressing a protein called CD69, but they also detected further upregulation of CD69 following infection. Considering that CD69 mediates the retention of T cells in both the genital tract and the lymph nodes, determining its role in the maintenance and spread of HIV infection will require further investigation.

The work of Ma et al. highlights the importance of studying all the individual compartments within the genital tract, and the need for HIV prevention strategies to target the whole tract, including the endometrium, and not just the lower tract. And given the diversity of T cells that can be infected, it is essential that strategies that involve increasing the number of T cells in various tissues for protection do not also increase the number of cells that can be targeted by the virus.

The study also raises interesting questions. How does the HIV susceptibility of T cells from the endometrium compare to that of T cells from the lower tract under these methodological conditions? Is endometrial infection particularly prone to spreading, or would the remodeling of T cells in the lower tract have a similar impact? Is remodeling influenced by the tissue environment and sex hormones? Does HIV remodel Th17 cells in the lower tract? Future studies comparing samples from the upper and lower tract from the same women are needed to answer these questions.
